# Supplementary material for: Doctors’ Professional and Personal Reflections: A Qualitative Exploration of Physicians’ Views and Coping during the COVID-19 Pandemic
Source: Int J Environ Res Public Health. 2023 Mar 24;20(7):5259. doi: 10.3390/ijerph20075259 (PMC10094024; doi:10.3390/ijerph20075259)
Supplement: Supplementary file 1 [file ijerph-20-05259-s001.zip › ijerph-2259839-supplementary.pdf]

Supplementary Material

Material S1

Semi-structured interview guide: Doctors' Professional and Personal Reflections: A Qualitative Exploration of Physicians' Views and Coping during the COVID-19 Pandemic

1. What does the origin of the COVID pandemic signify to you?

2. How is the experience of the pandemic affecting you personally and professionally?

3. What is your emotional experience related to this pandemic, in personal and professional circumstances?

4. What keeps you hopeful during this time? Any particular reasons, values, goals, or beliefs?

5. What lessons are you learning from the COVID pandemic? Specifically, to your professional life? How about personal life?

6. What will you do differently when this is over?

7. What should others learn from this? Think about your professional and personal experiences

Ending questions

1. How was the experience of this interview for you?
2. Did any questions bother you?
3. Did you like any questions?
4. Anything we missed?
